# Impact of Covid-19 pandemic on a general surgery clinic

**DOI:** 10.25122/jml-2022-0087

**Published:** 2022-03

**Authors:** Catalin Vladut Ionut Feier, Calin Muntean, Razvan Bardan, Andra Olariu, Sorin Olariu

**Affiliations:** 1. 1^st^ General Surgery Clinic, Pius Brinzeu Clinical Emergency Hospital, Timisoara, Romania; 2. First Discipline of Surgery, Victor Babes University of Medicine and Pharmacy, Timisoara, Romania; 3. Department of Informatics and Medical Biostatistics, Victor Babes University of Medicine and Pharmacy, Timisoara, Romania; 4. Department of Urology, Victor Babes University of Medicine and Pharmacy, Timisoara Romania; 5. Faculty of Medicine, Victor Babes University of Medicine and Pharmacy, Timisoara, Romania

**Keywords:** surgery clinic activity, monthly mortality rate, Covid-19, length of hospitalization

## Abstract

The Covid-19 pandemic has had a massive impact on global health, social and economic well-being worldwide. In addition to the direct effects of the disease on patients infected with the virus, this pandemic has severely affected the activity of surgical clinics around the world. One of the significant issues was an important decrease in the number of surgeries performed even in countries with highly performant medical systems. This study aimed to analyze the effects of the pandemic on the 1^st^ General Surgery Clinic in Timisoara County Hospital, compared to 2018–2019. In order to conduct this study, data regarding the activity of the clinic in the pandemic period and 2018–2019 was collected and analyzed from a statistical point of view, considering a p<0.05 as statistically significant. There were significant differences between the 2 periods regarding the number of hospitalized patients, the number of patients undergoing elective or emergency surgery, and the total number of surgeries performed. Due to the restrictions imposed, there was an increase in the average monthly mortality rate and a decrease in the average length of hospital stay. Covid-19 pandemic led to a severe activity restriction of the surgery clinics due to the restrictions imposed by the authorities and the reorganization of the clinics to comply with the epidemiological requirements. Also, the limitation of patients' access to surgical treatment and their fear of coming in contact with the hospital environment led to their presentation in more advanced stages of the disease, with more severe symptoms and a higher mortality rate during the pandemic.

## INTRODUCTION

The presence of the Sars CoV-2 RNA virus has been identified in China, and it has been spreading rapidly worldwide. Thus, on March 11, 2020, the World Health Organization (WHO) declared this disease a pandemic [1]. On February 26, 2020, the first case of Covid-19 was confirmed in Romania, and the government introduced the state of emergency on March 16. Furthermore, drastic government restrictions were applied to prevent the spread of the virus.

The impact of the pandemic on the health system and the practice of surgery was highlighted by the distribution of medical supplies and human resources to treat people with Sars CoV-2 virus infection, by increasing the number of intensive care beds, redistribution of medical and auxiliary staff to intensive care, procedural prioritization, and the restriction of the activity of surgical departments [2, 3].

The activity of surgical departments has always been related to the restrictions imposed by the authorities, so during the pandemic waves, when additional resources were needed to treat patients with Covid-19, the number of elective surgeries was significantly reduced. It should be noted that with the evolution of the pandemic and its waves, the percentage of elective interventions began to increase, mainly in the cases of patients with oncological diseases. The Covid-19 pandemic has immediate and long-term effects on millions of patients with surgical conditions, and it is expected to lead to additional requests for surgical services once this period ends [4, 5].

## MATERIAL AND METHODS

In order to carry out this study, several activity parameters of the First Clinic of General Surgery of the County Hospital Pius Brinzeu from Timisoara, Romania, were analyzed. The variation of these parameters in the period 26.02.2020–31.12.2021 was analyzed and compared to the same period of 2018–2019. Thus, the total number of hospitalized patients, the number of hospitalized patients for emergency treatment, and the number of hospitalized patients for elective treatment were analyzed. Also, the number of patients who underwent surgery in the two periods, the average monthly hospitalization duration, and the monthly mortality rate corresponding to the two periods of the study were analyzed. Finally, parameters such as the capacity of the surgical clinic (number of wards, number of beds) and the number of infected medical staff during the pandemic period were taken into account.

The data obtained were processed using IBM Spss Statistics for Windows. For the numerical data, the parameters of the central tendency (average, mode, median) and dispersion (standard deviation) were studied using statistical tests, where a p<0.05 was reported as statistically significant.

## RESULTS

The pandemic influence on the First Clinic of General Surgery of the County Hospital in Timisoara in 2020–2021 was analyzed and compared to the period 2018–2019. It should be mentioned that the 1^st^ General Surgery Clinic is one of the 3 Surgery Clinics of the County Hospital in Timisoara.

The capacity of the clinic where the study was performed is 17 wards. During the pandemic, 1 ward (5.88%) was reserved for performing PCR tests to detect the Sars CoV-2 virus, and another 3 wards (17.64%) were reserved for patients in the isolation area until the result of the PCR test.

Also, due to the imposed restrictions, the capacity of the wards was reduced by 50%, and given that wards were reserved for testing and isolating patients, the capacity of the clinic decreased by 56.25%.

During the pandemic, 2360 patients were hospitalized, and in the period 2018–2019, 3885 were hospitalized, observing a decrease in the number of patients by 39.25%. Their distribution according to the way of presentation and the type of surgical treatment (elective surgery or emergency surgery) are presented in Table 1.

**Table 1.** Distribution of patients in 2018–2019 compared to 2020–2021.

		2018–2019	2020–2021
**Total Patients**	Average (min-max)	161.87 (121–208)	98.33 (19–194)
	Standard Deviation	25.3	37
	Median	165	95.5
	p<0.001		
**Emergency**	Average (min-max)	60.5 (39–84)	39 (18–58)
	Standard Deviation	10.73	13.1
	Median	57	41
	p<0.001		
**Outpatient**	Average (min-max)	101.12 (64–140)	56.2 (1–137)
	Standard Deviation	23.05	31.38
	Median	105	60
	p<0.001		
**Surgeries performed**	Average (min-max)	141.25 (106–181)	85.29 (17–180)
	Standard Deviation	22.34	37.77
	Median	147.5	86.49
	p<0.001		

The evolution of daily infections during the pandemic was analyzed considering pandemic waves. This study presents the impact of I-IV pandemic waves and their effects on the 1^st^ Clinic of General Surgery. The data presented are monthly centralized on each wave. The effects of these waves on the clinic are presented in Table 2.

**Table 2.** Distribution of patients considering the waves of Covid-19 pandemic.

		1^st^ Wave	2^nd^ Wave	3^rd^ Wave	4^th^ Wave
**Total patients**	Average (min-max)	61 (19–119)	78.75 (65–93)	97.25 (95–99)	105
	Standard Deviation	51.88	15.32	2.06	21.7
	Median	45	78	97	94
**Emergency**	Average (min-max)	37 (18–54)	36.25 (29–48)	33 (27–37)	43.33 (39–48)
	Standard Deviation	18.08	8.22	4.89	4.5
	Median	39	34	34	43
**Outpatient**	Average (min-max)	24 (1–65)	42.49 (30–64)	64.25 (58–69)	61.66 (51–82)
	Standard Deviation	35.59	15.37	5.2	17.61
	Median	6	38	65	52
**Surgeries performed**	Average (min-max)	53 (17–102)	70.74 (55–85)	87.75 (84–90)	95.33 (78–121)
	Standard Deviation	44.67	15.06	2.63	22.68
	Median	39	71	86	87

The variation of the monthly mortality rate in the clinic during the pandemic was analyzed and compared to the period 2018–2019 in Table 3.

**Table 3.** Monthly average mortality rate in 2018-2019 compared to 2020-2021.

		2018–2019	2020–2021
**Length of average hospital stay**	Average (min-max)	7.72 (6.51–9.38)	6.75 (5.18–9.78)
	Standard Deviation	0.82	1.18
	Median	7	6
	P=0.004		
**Mortality rate**	Average (min-max)	3.62 (1.49–6.15)	5.34 (2–12.31)
	Standard Deviation	1.31	2.97
	Median	3.38	4.4
	P=0.02		

## DISCUSSION

The pandemic caused by the Sars CoV-2 virus has had a major impact on the management of healthcare systems worldwide, immediately becoming a global public health issue. In Romania, the first case of Covid-19 was confirmed on 26.02.2020, and the state of emergency was introduced on 16.03.2020 [6]. Healthcare systems worldwide have been overwhelmed by the massive number of daily infections, and the capacity of intensive care units was rapidly overwhelmed even in countries with high-performance healthcare systems such as Germany [7]. This global health crisis led to an increase in the necessity of intensive care beds and redistribution of staff in these wards. Regarding the surgical clinics, the start of the pandemic generated massive changes in their surgical activity by drastically reducing the number of surgeries performed and the capacity of the clinic to comply with epidemiological norms [8].

An essential aspect worth considering is the attitude of the citizens towards the restrictions imposed by the authorities and towards the fear of contacting the new coronavirus. Thus, patients were advised not to go to the health units except in case of severe symptoms to reduce as much as possible unnecessary visits and contact with other people.

Also, an important factor in the decrease of patients who presented to the hospital was their fear of coming into contact with an environment where there was a possibility of interacting with infected people.

The capacity of the First Clinic of General Surgery was also influenced by the Covid-19 pandemic. Thus, out of the 17 wards from the section, 4 (23.53%) were redistributed as follows: one ward (5.88%) was selected for testing people to detect Sars CoV-2 virus infection, and 3 wards (17.64) were assigned to isolate patients for 24 hours until PCR test results arrived. This decrease in the capacity of surgical clinics was reported in other countries from Europe, restrictions being applied to cope with the epidemiological norms [9].

Following the mentioned modifications, in the remaining 13 wards, the number of available beds was halved. There was a decrease in the capacity of the clinic by 59.37% compared to the 2018–2019 period. It is worth mentioning that more than a quarter of the clinic surgeons presented a history of Covid-19 during the pandemic. In terms of nurses, this percentage increased to 33.33%, and in the case of auxiliary staff to 56.25%. Some studies show that almost 5 to 7% of doctors were infected with coronavirus and that the auxiliary personnel got an even higher infection rate. It was also shown that the auxiliary health workers were infected at a larger rate due to the duties performed, including close contact with patients and a higher degree of socialization in the hospital between them [10].

Within the 1^st^ General Surgery Clinic of Timisoara County Hospital, there was a decrease of 39.25% in the total number of hospitalized patients during the pandemic compared to 2018–2019. This was due to the restrictions imposed by the authorities on the surgical departments and the recommendations to visit the sanitary units only in emergency cases. Patients' fears must also be taken into account.

In the first part of the Covid-19 pandemic, the number of hospitalized patients was seriously reduced. The authorities imposed restrictions on surgeons' activities. They were allowed to perform mainly emergency interventions. The type of interventions performed as a rule in the 1^st^ General Clinic varied a lot from treating perianal abscess to the treatment of colon cancer, or inguinal hernia treatment, to amputation etc.

There were a maximum of 194 hospitalized patients in a month and a minimum of 19 during the pandemic (this was achieved at the beginning of the pandemic in Romania when government restrictions were among the most severe). There were significant differences between the pandemic and 2018–2019 periods (p<0.001), highlighting extremely significant differences between the two periods regarding the number of hospitalized patients.

The monthly average of emergency hospitalized patients decreased by 35.5% during the pandemic (p<0.001), highlighting extremely significant differences between the number of emergency hospitalized patients between the two periods. A massive reduction of non-urgent surgical activity was reported worldwide [11] during the pandemic, showing statistically significant differences between the pandemic period and the previous period, the decrease having a variation from 17% in New Zealand [12] to over 48% in Spain [13].

There was a 44.42% decrease in the number of patients hospitalized for elective surgery during the pandemic (p<0.001), highlighting extremely significant differences between the two periods.

Due to the restrictions imposed and the need to comply with epidemiological norms, the total number of surgeries performed during the pandemic was significantly lower (p<0.001), showing extremely significant differences between the two periods, with a decrease of 39.61% in the number of interventions performed.

The 4 parameters presented above (total number of hospitalized patients, total number of hospitalized patients for elective treatment, total number of emergency hospitalized patients, total number of surgeries performed) were influenced by the Covid-19 pandemic. The decrease in these parameters was due to restrictions imposed by authorities, advising patients not to visit the health units except in case of severe symptoms, as well as their fear of visiting hospitals, but also due to adjustments made in the ward to comply with epidemiological norms and therefore reduce its capacity [9, 14, 15].

Changes were reported in Asia as well. For example, China, the start point of this pandemic, reported a decreased rate of 35% for patients undergoing elective surgery; India was no exception, showing a decrease of 42.4% [16, 17].

According to Table 2, there is an increase in the average number of patients admitted for elective treatment or emergency department and an increase in the number of surgeries performed in the clinic, associated with the evolution of the pandemic and its waves. This is directly related to the severity of the restrictions applied to the population and the surgery clinic. Following the analysis of the wave period compared to the corresponding period of 2018–2019, multiple p-values<0.05 were obtained, which show a difference between the number of patients hospitalized in the clinic during the pandemic waves, compared to the same periods of 2018–2019.

After analyzing the average length of hospitalization and the monthly mortality rate within the ward, differences were highlighted during the pandemic compared to the previous period. Thus, a shortening of the average duration of hospitalization during the pandemic was observed (p=0.004), showing very significant differences between the two periods. In order to limit the contact between the patients and the surgeons or auxiliary staff, the patients were discharged more quickly, once they did not present postoperative complications and were hemodynamically stable, appetizing, feverish, with intestinal transit present, with the indication to return in case of noticeable symptoms. In a large study conducted on 2073 cases in 40 countries, the average length of hospital stay decreased from 7 days (min.5–max.10) before the pandemic to 6 days (min.4–max.8) (p<0.001), showing that there were extremely significant differences between the two periods, indicating the tendency of shortening the average hospital stay of patients [18].

Due to the restrictions imposed and the patients' fear of visiting the hospital units, they presented to the hospital with severe symptoms, with more advanced pathologies, or requiring emergency treatment, which inevitably led to an increase in the mortality rate. The monthly mortality rate in the clinic increased by 47.51% compared to the pre-pandemic period (p=0.02), which shows the presence of significant differences between the two periods.

In another study performed in this clinic, when analyzing the pandemic period compared to the previous period regarding the presence of 90 days postoperative mortality among patients undergoing surgery for colon cancer treatment, there was an increase from 11.3% in 2028–2019 to 24.5% in the pandemic period, with significant differences between the two periods (p=0.029).

## CONCLUSION

The Covid-19 pandemic has affected the health system worldwide, becoming a global public health problem. The activity of the surgery departments was massively influenced by the restrictions imposed on the population and the organizational procedures of the surgery clinics.

The total number of patients who were hospitalized decreased significantly compared to the period before the pandemic (p<0.001), this decrease was observed both among patients who underwent elective treatment (p<0.001) and among patients who had emergency surgical treatment (p<0.001). In close connection with these parameters, the total number of surgeries performed was also influenced (p<0.001).

Taking into account the higher risk of infection in hospital units, the surgeons preferred to shorten the average period of hospitalization, which decreased during the pandemic showing a statistical significance (p=0.004), along with the desire of patients to leave faster due to the fear of becoming infected with the new coronavirus. Due to the presentation of patients in more advanced stages of the disease and more severe symptoms, the mortality rate increased significantly (p=0.02) compared to the pandemic period and the period 2018–2019.

## ACKNOWLEDGMENTS

### Conflict of interest

The authors declare no conflict of interest.

### Ethical approval

The data collection was done after obtaining the approval of the Ethics Commission of the Timisoara County Hospital (approval number: 282/09.02.2022).

### Authorship

CVIF, CM, and SO contributed to conceptualization. CVIF, AO, SO contributed to the methodology section. CVIF contributed to the software. CVIF, CM and SO contributed to validation. AO,SO contributed to formal analysis. CVIF, AO, and SO contributed to the investigation. CVIF, CM, AO and SO contributed to resources. CVIF contributed to data curation. CVIF, SO contributed to writing – original draft preparation. CVIF, CM and SO contributed to writing, reviewing, and editing. CVIF and SO contribute to visualization. SO contributed to supervision and CVIF to project administration.
